# Notes

**Published:** 1898-05

**Authors:** 


### Notes.

### SAJOUS'S ANNUAL AND ANALYTICAL CYCLOPÆDIA OF PRACTICAL MEDICINE.

CLOTH, $5.00; HALF RUSSIA, $6.00

The F. A. Davis Company, Publishers, Philadelphia.

For years there has been a constant and increasing chasm between the generally-accepted facts in medicine and surgery and that which is more or less subject to debate. There was once a happy time, years ago, when the average doctor learned his trade and practiced it in peace and quiet thereafter. But those days have gone, never to return.

The Americans—perniciously active, according to our European friends, in their methods of turning to account any scientific discovery—have left no stones unturned in attempts to realize practical advantage from every discovery in medicine and surgery, no matter from what source it might originate.

A great relief was afforded by Sajous's Annual in classifying the periodical literature, especially to those who have taken each consecutive issue; but there is nothing so good that it cannot be improved upon; and thus, after many years of careful consideration, the editor has worked out what is believed to be the solution of the whole problem.

Dr. Sajous—whose experience, all will admit, has been sufficient to constitute him a person qualified to judge—believes it is absolutely feasible to combine the features of a text-book and of a work like Sajous's Annual in one system. His idea has been submitted to many eminent medical writers and has, in every instance, received their unqualified approval. Likewise a large number of busy, general practitioners, who not only feel the financial tax for medical works quite severely, but find that the possession of a large reference library only entails a corresponding amount of labor in using it, look upon Dr. Sajous's plan with particular favor.

The new publication has the alphabetical arrangement, and comprises a concise statement of the generally-accepted methods in vogue, in one style of type, while in a different type, on the same page, can be found the opinions of well-known authorities bearing upon whatever may be debatable regarding the subject in hand. This alphabetical arrangement will consider all the practical subjects of medicine and surgery and the clinical application of therapeutics. It will appear at the approximate rate of one volume each six months, the whole alphabet being thus covered in three years, and during this time a monthly supplement (The Monthly Cyclopædia), alphabetical from A to Z, will be brought out; so that a doctor can have a complete synopsis of the latest journal literature to reinforce his system of reference.

Subscriptions are taken for the entire series only, at $5.00 per volume, in cloth. This secures a large six-volume reference system with thirty-six monthly supplements during that period.

The volumes will be beautifully illustrated; each volume will be handsomely bound in two colors of cloth. The half Russia binding will be furnished at $6.00 per volume, if desired.

It will thus be seen that the aim of Dr. Sajous and his editorial staff is designed to accomplish two things: 1st. To give a satisfactory statement of what may be safely relied upon as the best general method of treatment in any given case. 2d. To combine with this a means of practically utilizing the discussion by the leading medical authorities of the world which may in any degree modify present, established methods.

---

A meeting of the Alumni Association of the Southern Medical College is called for the 20th, 21st, and 22d of July, 1898. For further information, write to the undersigned or to the secrerary, Dr. J. R. Shannon, Cabaniss, Ga. Railroad rates one-fifth fare on all roads, due to the meeting of the Confederate Veterans' Reunion.

J. McF. GASTON, JR., A.M., M.D., President.

---

We are informed that Dr. J. McFadden Gaston has within the past month resigned the professorship of principles and practice of surgery in the Southern Medical College.

This step was contemplated a year ago by Dr. Gaston, and he was induced to postpone it at the urgent solicitation of some of his colleagues in the faculty.

He expects in future to devote a large share of attention to the treatment of disorders by electricity, and will open an office in the new Prudential building for the purpose. Dr. J. McF. Gaston, Jr., has also resigned his position in the Southern Medical College, and will be associated with his father in the future, as he has been in the past, in the firm of Drs. Gaston & Gaston.

